# Development of prognostic models for survival and care status in sporadic Creutzfeldt-Jakob disease

**DOI:** 10.1093/braincomms/fcac201

**Published:** 2022-08-02

**Authors:** Akın Nihat, Janice M Ranson, Dominique Harris, Kirsty McNiven, TzeHow Mok, Peter Rudge, John Collinge, David J Llewellyn, Simon Mead

**Affiliations:** MRC Prion Unit at UCL, UCL Institute of Prion Diseases, Cleveland Street, London W1W 7FF, UK; National Prion Clinic, National Hospital for Neurology and Neurosurgery, University College London Hospitals NHS Foundation Trust, London WC1N 3BG, UK; College of Medicine and Health, University of Exeter, Exeter EX1 2HZ, UK; Deep Dementia Phenotyping Network, Exeter EX1 2LU, UK; MRC Prion Unit at UCL, UCL Institute of Prion Diseases, Cleveland Street, London W1W 7FF, UK; National Prion Clinic, National Hospital for Neurology and Neurosurgery, University College London Hospitals NHS Foundation Trust, London WC1N 3BG, UK; MRC Prion Unit at UCL, UCL Institute of Prion Diseases, Cleveland Street, London W1W 7FF, UK; National Prion Clinic, National Hospital for Neurology and Neurosurgery, University College London Hospitals NHS Foundation Trust, London WC1N 3BG, UK; MRC Prion Unit at UCL, UCL Institute of Prion Diseases, Cleveland Street, London W1W 7FF, UK; MRC Prion Unit at UCL, UCL Institute of Prion Diseases, Cleveland Street, London W1W 7FF, UK; National Prion Clinic, National Hospital for Neurology and Neurosurgery, University College London Hospitals NHS Foundation Trust, London WC1N 3BG, UK; College of Medicine and Health, University of Exeter, Exeter EX1 2HZ, UK; Deep Dementia Phenotyping Network, Exeter EX1 2LU, UK; Alan Turing Institute, London NW1 2DB, UK; MRC Prion Unit at UCL, UCL Institute of Prion Diseases, Cleveland Street, London W1W 7FF, UK; National Prion Clinic, National Hospital for Neurology and Neurosurgery, University College London Hospitals NHS Foundation Trust, London WC1N 3BG, UK

**Keywords:** prion, dementia, prognosis, survival model, Creutzfeldt-Jakob

## Abstract

Sporadic Creutzfeldt-Jakob disease, the most common human prion disease, typically presents as a rapidly progressive dementia and has a highly variable prognosis. Despite this heterogeneity, clinicians need to give timely advice on likely prognosis and care needs. No prognostic models have been developed that predict survival or time to increased care status from the point of diagnosis. We aimed to develop clinically useful prognostic models with data from a large prospective observational cohort study. Five hundred and thirty-seven patients were visited by mobile teams of doctors and nurses from the National Health Service National Prion Clinic within 5 days of notification of a suspected diagnosis of sporadic Creutzfeldt-Jakob disease, enrolled to the study between October 2008 and March 2020, and followed up until November 2020. Prediction of survival over 10-, 30- and 100-day periods was the main outcome. Escalation of care status over the same time periods was a secondary outcome for a subsample of 113 patients with low care status at initial assessment. Two hundred and eighty (52.1%) patients were female and the median age was 67.2 (interquartile range 10.5) years. Median survival from initial assessment was 24 days (range 0–1633); 414 patients died within 100 days (77%). Ten variables were included in the final prediction models: sex; days since symptom onset; baseline care status; *PRNP* codon 129 genotype; Medical Research Council Prion Disease Rating Scale, Motor and Cognitive Examination Scales; count of MRI abnormalities; Mini-Mental State Examination score and categorical disease phenotype. The strongest predictor was *PRNP* codon 129 genotype (odds ratio 6.65 for methionine homozygous compared with methionine-valine heterozygous; 95% confidence interval 3.02–14.68 for 30-day mortality). Of 113 patients with lower care status at initial assessment, 88 (78%) had escalated care status within 100 days, with a median of 35 days. Area under the curve for models predicting outcomes within 10, 30 and 100 days was 0.94, 0.92 and 0.91 for survival, and 0.87, 0.87 and 0.95 for care status escalation, respectively. Models without *PRNP* codon 129 genotype, which is not immediately available at initial assessment, were also highly accurate. We have developed a model that can accurately predict survival and care status escalation in sporadic Creutzfeldt-Jakob disease patients using clinical, imaging and genetic data routinely available in a specialist national referral service. The utility and generalizability of these models to other settings could be prospectively evaluated when recruiting to clinical trials and providing clinical care.

## Teaser

Nihat and Ranson *et al*. report the development of accurate prognostic models in sporadic Creutzfeldt-Jakob disease for survival and care needs escalation. The inputs to the models are readily available clinical scales and investigations that are available at a specialist diagnostic assessment. The models might be further developed and prospectively evaluated.

## Graphical Abstract

**Graphical Abstract:**
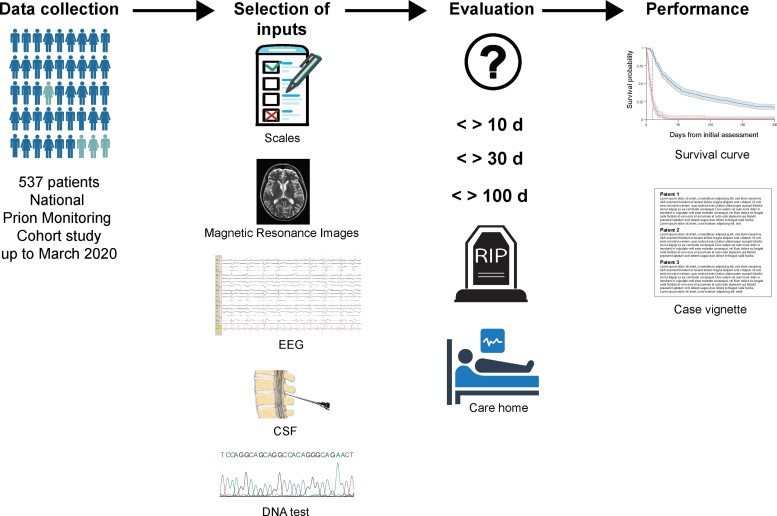

## Introduction

Sporadic Creutzfeldt-Jakob disease (sCJD) is a fatal neurodegenerative disease that manifests as a usually rapid, multidomain dementia associated with myoclonus, motor, and sensory impairments. Median survival from symptom onset is 5 months.^1^ Prognosis is highly variable and right-skewed in distribution, ranging from a few weeks to over 10 years.^2,3^ Whilst there is no treatment to cure or slow disease progression, appropriate patient management involves the avoidance of needless investigations and timely care planning.^4,5^ In the UK, patients are often investigated and diagnosed during a hospital admission. Potential discharge options include hospice care, another 24-h care facility, home care-package, or no/informal care at home. These decisions are informed by clinical judgement of likely prognosis and the speed of clinical decline anticipated. Accurate prognostication and prediction of care status are therefore crucial for provision of optimal and timely support.

Human prion diseases, of which sCJD is the most common, involve the templated misfolding of cellular prion protein into disease-associated assemblies, including forms that propagate by fission and forms that cause neurodegeneration.^6^ Prion diseases are associated with propagation of prion strains, which lead to distinct clinical and pathological phenotypes, which are maintained on transmission to other humans or animals and are encoded by the molecular structure of prions.^6^ The prion protein gene (*PRNP*) is polymorphic in many populations, with a common variation being found at Codon 129, encoding amino acids methionine or valine. Numerous clinicopathological and molecular studies have shown the codon 129 genotype as a key determinant of survival, rate of progression, and clinical phenotype, in part related to conformational selection of permissible prion strains.^1,7,8^ Several demographic, protein biofluid, brain imaging, and neurophysiological biomarkers are associated with prognosis or subtypes of sCJD.^1,9–14^ We and others have evaluated several tools to measure patient progression.^3,15^ This knowledge provides a rational basis to improve prognostication using multivariable modelling.

Previous studies have had some success in predicting survival but have significant limitations. Staffaroni *et al*.^16^ identified CSF biomarkers associated with survival and Sundaram *et al.*^17^ used a neuropsychological assessment score to predict probability of 3-, 6- and 12-month survival though neither tested combinations of predictors. Llorens *et al*. developed a prognostic model of 6-month survival from sCJD symptom onset incorporating age, sex, *PRNP* codon 129 genotype, and CSF tau, which whilst valuable, did not model survival from the point of clinical decision making. Model performance was moderate [area under the curve = 0.686; 95% confidence interval (CI), 0.67–0.71], and they did not produce a prognostic model for escalating care status.^18^ Here, we aimed to develop accurate individual prognostic models for survival from the point of a clinical assessment, and separately model care need progression using a wide range of prospective clinical, imaging, genetic and biomarker data from the UK’s National Prion Monitoring Cohort study.

## Methods

Reporting of our study follows the Transparent Reporting of a multivariable prediction model for Individual Prognosis or Diagnosis (TRIPOD) statement^19^ (see the TRIPOD checklist in Supplementary methods).

### Data and study population

We used longitudinal data on 537 patients with sCJD from the National Prion Monitoring Cohort (Cohort study) described in detail elsewhere.^3^ The sample comprised all patients with sCJD enrolled in the Cohort study in March 2020. The Cohort study is a UK-wide observational study of human prion diseases led by the NHS National Prion Clinic, which prospectively collects data from patients as part of a clinical assessment including demographics, key investigation results, imaging, prion protein gene sequencing, and bespoke clinimetric scales.^3,15^

A national referral system for prion diseases was set up in the UK in 2004. UK neurologists were asked by the Chief Medical Officer to refer all patients with suspected prion disease jointly to the National CJD Research and Surveillance Unit (Edinburgh, UK) and to the NHS National Prion Clinic (London, UK) for the purpose of both epidemiological surveillance, provision of specialist clinical care and also participation in clinical research, including clinical trials and the Cohort study. The Cohort study began in October 2008, and aimed to enrol all symptomatic patients with prion disease in the UK thereafter. The Cohort study collected natural history data very similar to that used in the Medical Research Council (MRC) PRION1 trial of quinacrine 2004–08, and merged with the natural history data from this trial.^20^ In 2015, in order to focus on the natural history of patients who were not already moribund, the protocol was altered such that patients with high levels of neurodisability and MRC Scale < 5 at initial assessment were no longer eligible. The Cohort study team visit patients within 5 working days of notification. For the purposes of this report, the initial study visit was assumed to be the point of diagnosis. In reality, it is difficult to define a precise point of diagnosis of sCJD as evidence typically accrues over a period of a few weeks in-patient care, based on a combination of the clinical features and results of MRI brain (done at a median 3 weeks before study visit (Q1–Q3 2–5 weeks), CSF, and EEG investigations. Patients were diagnosed according to contemporary diagnostic criteria with very high specificity.^21^ In the absence of disease-modifying therapeutics, only supportive symptomatic treatment, for example treatments for psychiatric disturbance, myoclonus, sleep disorder and end of life care, was given. Post-mortem examination was conducted in 241 of 504 (48%) patients who died during follow-up. Diagnostic accuracy was confirmed in all cases.

### Outcome variables

#### Survival

The primary outcome variable was death (yes/no) by three time-points: 10 days, 30 days and 100 days from first assessment. These outcomes were decided based on an expectation of judgements to be made by the treating physician: 10-day survival implies immediate end-of-life care, in the UK typically hospice care; whereas 100-day survival implies informal care, set up of an external care-package at home or consideration of nursing home care.

#### Increased care status

The secondary outcome variable was increased care status (yes/no), investigated in a subsample of 113 patients who at the time of initial assessment had lower care status [either no care, informal care (for example, some support from a family member), or formal care up to two times daily] and had a date recorded for progression to increased care status (formal care more than twice daily, or nursing home/hospice placement). This secondary outcome was assessed at the same time-points as the primary outcome: 10 days, 30 days and 100 days from first assessment.

### Predictor variables

A scoping literature review and expert panel discussion identified 29 variables associated or potentially associated with prion disease progression or prognosis. The review prioritized research evidence of an association between a predictor and CJD survival, rather than factors known to influence dementia survival more generally,^22^ and we excluded variables that, during the study period, were not feasibly available at diagnosis [serum tau, serum neurofilament light (NfL), prion molecular strain type (combination of *PRNP* codon 129 genotype and western blot type)], and those symptoms that may be a very significant burden for carers but fluctuate over short periods of time, or are treatable (e.g. myoclonus, psychiatric disturbance); 25 potential predictors remained. To reduce this further, the list of variables was independently rated by two clinicians with sCJD assessment expertise (A.N. and S.M., see Supplementary Methods 2), resulting in 13 candidate predictors for analysis.

#### Sociodemographics

Sex (male/female), age (years) and days since symptom onset were based on patient or relative reports.

#### Clinical assessments

The MRC Prion Disease Rating Scale score (MRC Scale; 0–20)^3^ is a functional composite measure of sCJD disease progression developed using item-response modelling. The Prion Disease Motor Scale (0–100)^15^ and Cognitive Scale (0–100)^15^ were later developed to measure progression of motor and cognitive impairments using a similar methodology. Mini-Mental State Examination (MMSE) total score (0–30)^23^ was also recorded. The clinical phenotype of sCJD was based on clinical judgement of the dominant symptomatic presentation as one of seven categories; ‘visual’, ‘ataxic’, ‘cognitive’, ‘psychiatric/behavioural’, ‘sleep/thalamic’, ‘stroke-like’ and ‘classical’.^24^ Due to the small number of patients in some categories we collapsed this to a three-category clinical phenotype variable of ‘classical’, ‘cognitive, psychiatric or behavioural’, and ‘other’. Care status was recorded for all assessments based on the following scale: admission to nursing home or hospice (0), formal care three/four times per day (‘all care’) (1), formal care up to twice per day (2), informal care (e.g. spouse, other relative or friend, not local authority or hospital care) (3), or no care (4). Data were collected between October 2008 and November 2020, with a total follow-up of 161 patient-years. For the primary analyses, due to the small number of patients in some of the care status categories we combined categories 1, 2, 3 and 4 to produce a binary variable of ‘nursing home or hospice care’ (yes/no). The secondary analyses predicting care status progression to Category 0 or 1 (higher care status), was conducted using data on 113 patients with baseline care status Category 2, 3 or 4 (lower care status). We used a binary variable representing formal care status at baseline (yes/no), defined as Category 2 versus Category 3 or 4.

#### Imaging

Local diagnostic diffusion-weighted MRI already available at the time of first assessment was used to assess the presence of five abnormalities by the recruiting neurologist (each yes/no); atrophy, cortical ribboning, pulvinar sign, basal ganglia and thalamic, as assessed by the study team. These were modelled as a single count variable (possible range 0–5). Necessarily this involved a range of scanner manufacturers and protocols as only a small proportion of sCJD patients are able to transfer to a regional or national specialist site for research investigations; given the wide geographical distribution of sCJD patients throughout the UK and absence of phenotypic clusters, we reasoned that any variation in imaging quality or protocol between referring hospitals would be random, and not bias results.

#### Genetics

*PRNP* codon 129 genotype (MM, MV or VV) was determined by *PRNP* sequencing.^25^ This was a special case variable in this study. Currently, this is not readily available at the first specialist diagnostic assessment. This variable was included as a candidate predictor as the genetic data are collected during the baseline assessment, via peripheral blood sample. However, it is worth noting that the results confirming *PRNP* codon 129 genotype currently take from several days to weeks to obtain in clinical practice.

#### Biomarkers

Presence of electroencephalography Periodic Sharp Wave Complexes was determined by reference to the local neurophysiology report of EEG available at first assessment. CSF s100b values (normal/abnormal) were determined by local reports with a cutoff of <0.42 ng/l.

### Data analysis

All analyses were performed using STATA version 16.0, including installation of the additional packages: st0177, mim, mfpmi, st0569, diagt, missings, st0139_1, looclass.

#### Missing data

Multivariate imputation by chained equations was conducted for missing data on all predictors according to guidance.^26^ Eighty imputed datasets were created, this number being greater than the percentage of cases with incomplete data (79%). Analyses were restricted to those with complete data on the outcome variables. Predictive mean matching to three nearest neighbours was used for continuous variables, multinomial logistic regression for categorical variables, ordinal logistic regression for ordered categorical variables and logistic regression for binary variables. Variables were imputed iteratively, from the one with the least missing data to the most. Age at enrolment, sex and survival outcomes was included in the imputation model as complete auxiliary variables.

#### Primary outcome model development

For the primary outcome of survival, prediction models were developed using a multivariable fractional polynomial approach.^27^ For each time-window, logistic regression using backwards elimination (BE) with a rejection criterion of *P* ≥ 0.05 was conducted on 1000 random bootstrap replication samples with replacement. In each replication, BE was combined with a closed test function selection procedure (FSP). FSP is a systematic search for non-linearity that investigates the most appropriate functional form for each continuous predictor, considering first- and second-order fractional polynomial terms to four degrees of freedom. This procedure ensures that variables are not erroneously disregarded due to inappropriate modelling of non-linear associations. To avoid overfitting, linear terms are used to model associations between continuous predictors and the outcome unless a more complex term provides a significantly better fit (*α* = 0.05).

#### Selection of predictors

Predictors were selected based on the proportion of times they were selected by the BE regression model in the bootstrapped samples. To aid clinical utility, we selected a single set of predictors for use in the final models predicting 10-, 30- and 100-day mortality. Variables with a bootstrap inclusion fraction (BIF) of 50% or more resulting from the *mfpboot* analysis for any of the three time-points were selected for inclusion in all three final models. Sensitivity analyses were conducted comparing model fit with and without each of the borderline variables not selected for the final models (borderline variables were defined as having a BIF between 25 and 49%).

#### Secondary outcome model development

Variables selected in the final model for the primary analyses were used to predict increased care status. Final models predicting increased care status were developed using the same approach as above, for the same three time-points: 10 days, 30 days and 100 days from first assessment.

#### Estimation of the final predictive models

Six multivariable fractional polynomial logistic regression models were estimated in the multiply imputed data, predicting 10-, 30- and 100-day mortality, and 10-, 30- and 100-day increased care status. The most appropriate functional form of continuous predictors was identified independently in each final model using the FSP described above. Predicted probabilities were calculated based on the model coefficients and averaged across imputations.

#### Evaluation of model performance

Receiver operating characteristic (ROC) curves and areas under the curve (AUC) were used to evaluate the discrimination performance of each of the six models. For each model, a binary classifier was generated using a predicted probability threshold of 0.5 (assuming equal importance of false-positives and false-negatives). This classifier was used to calculate rates of true-positives, true-negatives, false-positives and false-negatives, overall percentage correctly classified, and model performance statistics including sensitivity, specificity, positive predictive values and negative predictive values. In the absence of a suitable external validation dataset to estimate the generalizability of the models due to the rarity of sCJD, we repeated the model evaluation using leave-one-out cross-validation. This procedure involves estimating the model on *n*-1 observations, and applying the resulting prediction to the excluded observation. This was repeated excluding each observation in the data, and results were combined to generate the overall cross-validated model performance statistics. Finally, for the primary outcome, we plotted the actual survival of patients stratified by whether they were at low or high risk of death within 10, 30 and 100 days. Low and high risk categories were defined using the same predicted probability threshold as all other model evaluation metrics above (low risk = 0.00–0.49, and high risk = 0.50–1.00 predicted probability). Survival was plotted using the STATA *sts graph* function.

### Data availability

Patient data from the National Prion Monitoring Cohort are owned by University College London. This patient data are not currently publicly available. For researchers interested in accessing this data, further information can be found at https://www.ucl.ac.uk/national-prion-clinic/national-prion-monitoring-cohort-npmc.

## Results

### Sample characteristics

Detailed characteristics of the clinical sample are shown in Table 1. The total sample (*N* = 537) had a median age of 67.16 [interquartile range (IQR) 10.53], 280 (52.1%) were females and 198 (36.9%) were resident in a nursing home or hospice at baseline. The median delay between carer-reported symptom onset and date of the initial diagnostic assessment was 130.8 days. Median survival from initial assessment was 24 days (IQR = 71, range 0–1633). The number of patients who died within 10, 30 and 100 days from initial assessment was 127 (23.7%), 293 (54.6%) and 414 (77.1%), respectively. Of those with lower care status at baseline who progressed to requiring increased care (*N* = 113), the median time until increased care status was 35 days (IQR = 73, range 2–251). The number of patients requiring increased care status within 10, 30 and 100 days was 20 (17.7%), 55 (48.7%) and 88 (77.9%), respectively.

**Table 1:** Baseline characteristics of study participants (*N* = 537)

	10-day mortality		30-day mortality		100-day mortality	
	Survived *N* = 410	Died *N* = 127	Survived *N* = 244	Died *N* = 293	Survived *N* = 123	Died *N* = 414
Age in years, median (IQR)	66.82 (10.04)	68.68 (11.68)	65.05 (11.16)	68.72 (10.85)	64.76 (11.46)	67.93 (10.52)
Female, *N* (%)	218 (53.17)	62 (48.82)	122 (50.00)	158 (53.92)	68 (55.28)	212 (51.21)
Days since onset, median (IQR)^a^	147.50 (179.00)	77.00 (61.00)	190 (187.5)	85 (86.00)	232.00 (162.00)	95.00 (119.00)
Nursing home/hospice care, *N* (%)	93 (22.68)	105 (82.68)	25 (10.25)	173 (59.04)	12 (9.76)	186 (44.93)
Codon 129 polymorphism^a^						
MM, *N* (%)	149 (36.34)	99 (77.95)	57 (23.36)	191 (65.19)	57 (23.36)	191 (65.19)
VV, *N* (%)	119 (29.02)	17 (13.39)	68 (27.87)	68 (23.21)	68 (27.87)	68 (23.21)
MV, *N* (%)	142 (34.63)	11 (8.66)	119 (48.77)	34 (11.60)	119 (48.77)	34 (11.60)
MRC score, median (IQR)^a^	9.00 (9.00)	1.00 (3.00)	11.00 (9.00)	2.00 (7.00)	13.00 (9.00)	4.00 (9.00)
Motor score, median (IQR)^a^	38.76 (38.02)	0.00 (6.00)	47.97 (25.84)	7.00 (33.28)	53.40 (22.49)	18.70 (39.43)
Cognitive score, median (IQR)^a^	28.26 (58.80)	0.00 (0.00)	45.94 (45.85)	0.00 (11.00)	48.14 (44.85)	1.00 (38.00)
EEG PSWCs, *N* (%)^a^	88 (21.46)	65 (51.18)	24 (9.84)	129 (44.03)	10 (8.13)	143 (34.54)
CSF s100b abnormality, *N* (%)^a^	226 (55.12)	65 (51.18)	125 (51.23)	166 (56.66)	64 (52.03)	227 (54.83)
MRI abnormalities, median (IQR)^a^	2 (2.00)	2 (2.00)	2.00 (2.00)	2.00 (2.00)	2.00 (2.00)	2.00 (2.00)
MMSE score, median (IQR)^a^	2 (15.00)	0 (0.00)	10.00 (19.00)	0.00 (0.00)	11.00 (20.00)	0.00 (8.00)
Clinical phenotype^a^						
Classical, *N* (%)	138 (33.66)	49 (38.58)	67 (27.46)	120 (40.96)	32 (26.02)	155 (37.44)
Cognitive, psychiatric, behavioural, *N* (%)	125 (30.49)	42 (33.07)	90 (36.89)	77 (26.28)	55 (44.72)	112 (27.05)
Other, *N* (%)	147 (35.85)	36 (28.35)	87 (35.66)	96 (32.76)	36 (29.27)	147 (35.51)

Clinical phenotype ‘other’ includes visual, ataxic, sleep/thalamic and stroke-like phenotypes. IQR = interquartile range; MRC, Medical Research Council; PSWC, Periodic Sharp Wave Complexes; MMSE, Mini-Mental State Examination.

a There was missing data on this variable. Descriptive statistics are reported for imputed data.

### Selection of predictors

Of the 13 candidate predictors, age, EEG and CSF s100b abnormality did not have a BIF ≥ 50% in any of the three analyses and were not selected for inclusion in the final model. Final models comprised the 10 remaining variables: sex; days since symptom onset; baseline care status; *PRNP* codon 129 genotype; MRC Prion Disease Rating, Motor and Cognitive Scales, count of MRI abnormalities; MMSE score and clinical phenotype. Of variables included in the final models, those with the highest proportion of missing data were clinical phenotype category (32.7% of patients), cognitive score (26%) and codon 129 (18%); all other variables were missing for <7% of patients.

### Primary models predicting survival (*N* = 537)

**Figure 1:**
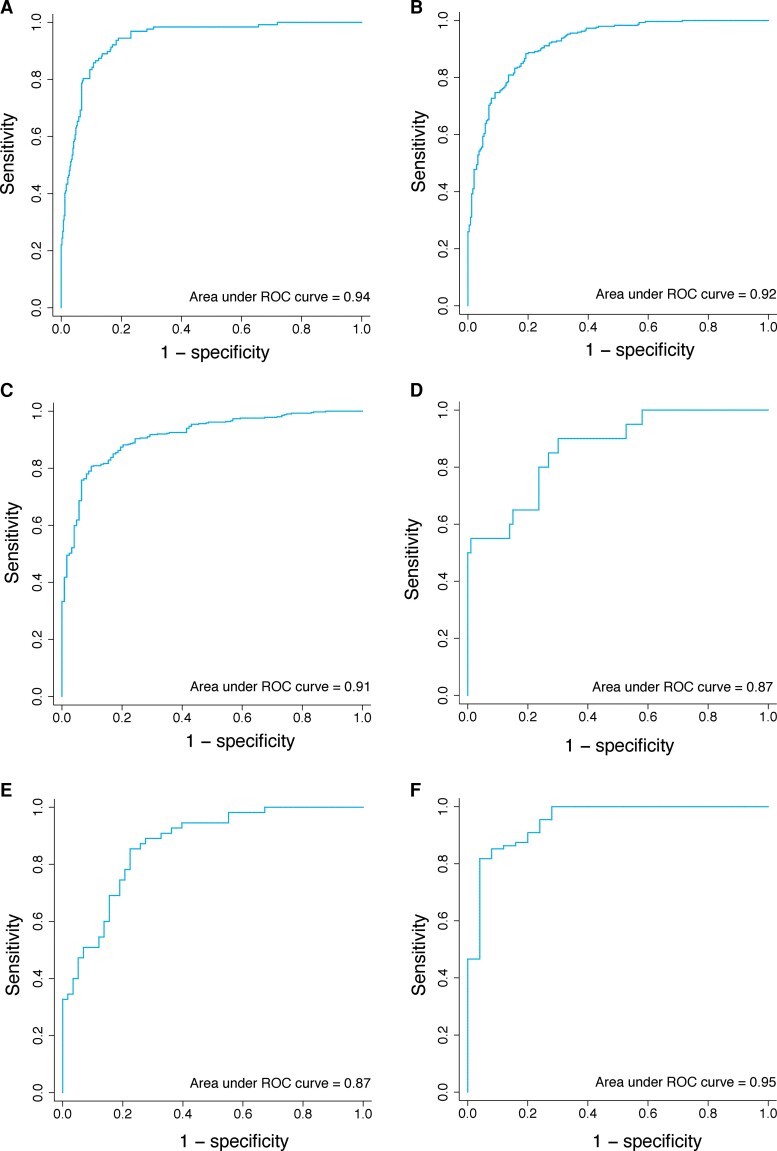
**ROC curves for primary models predicting mortality and secondary models predicting increased care status**. ROC curves for primary models (total patients = 537) predicting mortality within (**A**) 10 days, (**B**) 30 days, (**C**) 100 days, and for secondary models (total patients = 113) predicting increased care status within (**D**) 10 days, (**E**) 30 days and (**F**) 100 days. A threshold of 0.5 predicted probability is used to evaluate discrimination performance in each model.

**Figure 2:**
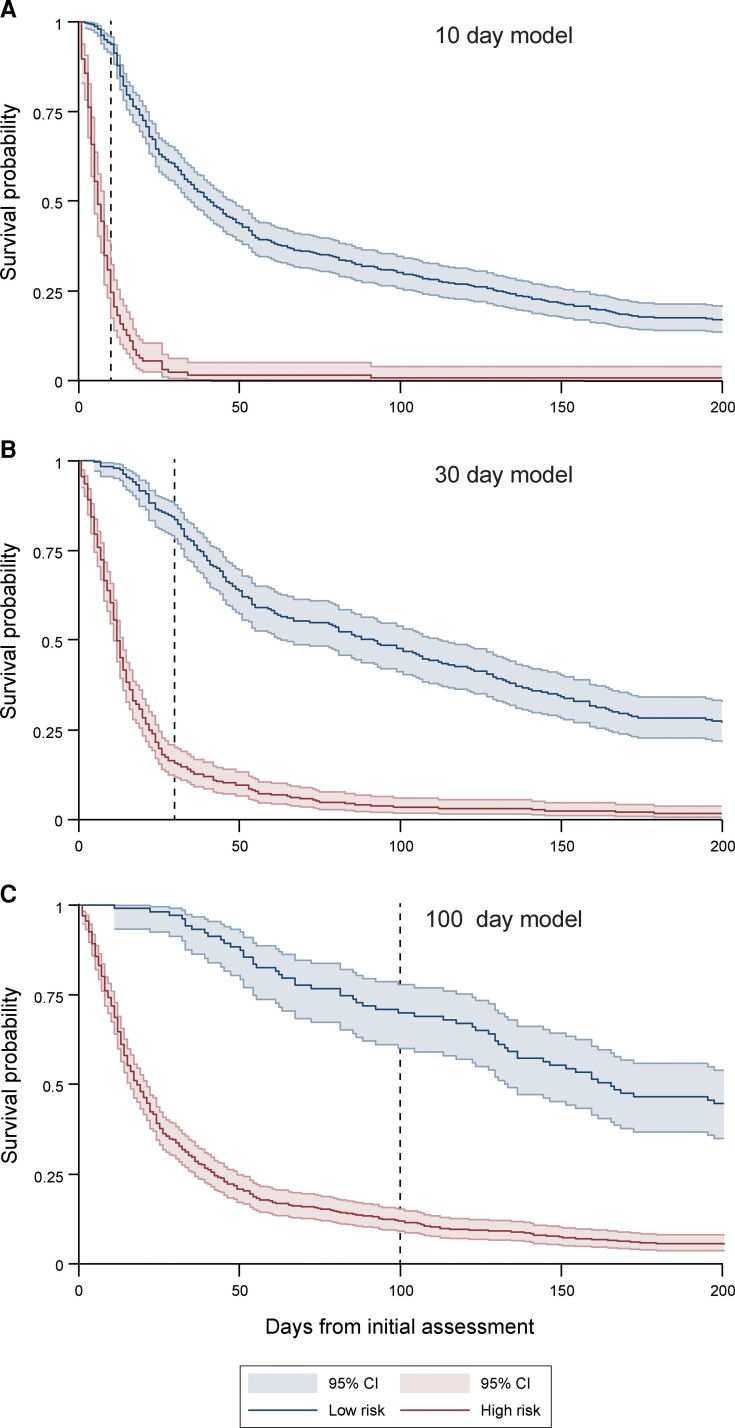
**Actual survival of patients stratified by model prediction of death**. Kaplan–Meier curves of actual patient survival when stratified as ‘high’ (red) or ‘low’ (blue) risk of death within (**A**) 10 days, (**B**) 30 days and (**C**) 100 days. Low-risk and high-risk groups were stratified by predicted probability of 0–0.49 and 0.50–1.00, respectively.

Final models predicting 10-, 30- and 100-day mortality are detailed in Supplementary Table 1. The overall percentage correctly classified by each model was 89.6, 84.2 and 84.7%, respectively. Classification rates and model performance statistics are presented in Table 2. All leave-one-out cross-validation results were within the 95% CI range estimated using the original development sample (see Supplementary Table 2). ROC curves for the primary models predicting survival are presented in Fig. 1. The actual survival of patients stratified by model predictions and 10, 30 and 100 days is shown in Fig. 2. Example patient vignettes are given in Box 1, illustrating care decisions made in specific anonymized patients and how these might have been influenced by the prognostic model.

Box 1 **Example patient vignettes illustrating decisions made for patients with a diagnosis of sCJD**

**Patient 1 (low risk <10 day; high risk <30 days)**: A lady in her seventh decade was referred to the National Prion Clinic with a 4-month history of progressive symptoms: initially visual misperceptions, hallucinations and achromatopsia. Two months after onset, she developed progressive episodic memory loss, dyspraxia, repetitive speech and a shuffling gait. At the time of review, she was a hospital inpatient for investigation. She had moderate global cognitive impairment, was almost cortically blind and unsteady, able to stand independently but required support for all daily activities. She had genotype MM at *PRNP* polymorphic codon 129. MRC Scale score was 11/20. Discharge planning was commenced, aiming for nursing or residential home placement, but the patient died 3 weeks later in hospital, before a suitable placement being identified. Model predictions may have helped the discharge team prioritize a hospice or urgent nursing home placement and avoiding her death in an acute hospital setting.

**Patient 2 (high risk <10 days)**: A lady in her eighth decade was referred with a 2-month history of progressive difficulty writing and performing basic arithmetic, blurred vision, dyspraxia, episodic memory loss, generalized myoclonus and Parkinsonism. On review, she was chairbound, had severe moderate cognitive impairment and required nursing support for all care. She had genotype MM at *PRNP* polymorphic codon 129. MRC Scale score was 8/20. After discussion with the patient’s family, an urgent hospice discharge was arranged within days; where she died a few days after transfer. Model predictions may have supported the discharge discussion.

**Patient 3 (low risk <10, <30 days; high risk <100 days)**: A man in his seventh decade was referred with a 5-month history of lethargy, insomnia and ataxia, followed by progressive episodic memory impairment and generalized myoclonus. On review by the National Prion Clinic, he had mild cognitive impairment particularly affecting executive function and attention. There was severe gait ataxia and a mild dysarthria, he could no longer mobilize and required support for sitting and dressing, but was otherwise not functionally impaired. Based on available information and expected prognosis, the patient and family were consulted and a decision reached to attempt discharge to his home. He had genotype VV at *PRNP* polymorphic codon 129. MRC Scale score was 16/20. Availability of appropriately trained carers and equipment provision was delayed after discharge, causing some distress to the patient’s family. His clinical condition progressed, and he died in hospital 40 days after review, during which time installation of some home equipment was still pending. Model predictions may have helped management of the discharge, being more aware that he likely only had a few months to live and if carers and equipment could not be organized promptly, then the home placement was no longer appropriate.

**Patient 4 (low risk <10, <30 days; high risk <100 days)**: A lady in her eighth decade was referred to the National Prion Clinic with 9 months of episodic memory impairment and unsteadiness, later developing paranoia, visual hallucinations, myoclonus and dysarthria. On review, she was bedbound with profound cognitive impairment, vocalized rarely in single words, had an impaired swallow and was incontinent of urine and faeces. She had genotype MV at *PRNP* polymorphic codon 129. MRC Scale score was 4/20. A hospice placement was initially mooted because of her advanced clinical stage. In fact, she was discharged to a care home and was well looked after for several months before she died. The decision to discharge to a care home rather than a hospice was probably a good judgement for this patient and would have been supported by model predictions.

**Table 2:** Classification rates and performance of models predicting 10-, 30- and 100-day mortality in sCJD patients (*N* = 537)

	Mortality within three time-points		
	10 days	30 days	100 days
True-positive	102 (19.0%)	254 (47.3%)	383 (71.3%)
False-positive	31 (5.8%)	46 (8.6%)	51 (9.5%)
True-negative	379 (70.6%)	198 (36.9%)	72 (13.4%)
False-negative	25 (4.7%)	39 (7.3%)	31 (5.8%)
Area under the curve (95% CI)	0.94 (0.92–0.96)	0.92 (0.90–0.94)	0.91 (0.89–0.94)
Sensitivity (95% CI)^a^	80.3% (72.3–86.8)	86.7% (82.3–90.4)	92.5% (89.5–94.9)
Specificity (95% CI)^a^	92.4% (89.4–94.8)	81.1% (75.7–85.9)	58.5% (49.3–67.3)
PPV (95% CI)^a^	76.7% (68.6–83.6)	84.7% (80.1–88.6)	88.2% (84.8–91.1)
NPV (95% CI)^a^	93.8% (91.0–96.0)	83.5% (78.2–88.0)	69.9% (60.1–78.5)

CI, confidence interval; PPV, positive predictive value; NPV, negative predictive value.

a Binary classifier using a 0.5 predicted probability threshold for each model.

### Secondary models predicting increased care status (*n* = 113)

Secondary models predicting 10-, 30- and 100-day increased care status are detailed in Supplementary Table 3. The overall percentage correctly classified by each model was 90.3, 77.9 and 90.2%, respectively. Classification rates and model performance statistics are presented in Table 3. All leave-one-out cross-validation results were within the 95% CI range estimated using the original development sample (see Supplementary Table 4). ROC curves for secondary models predicting increased care status are presented in Fig. 1.

**Table 3:** Classification rates and performance of models predicting 10-, 30- and 100-day increased care status in sCJD patients with lower care status at baseline (*N* = 113)

	Progression to increased care status within three time-points		
	10 days	30 days	100 days
True-positive	10 (8.9%)	42 (37.2%)	84 (74.3%)
False-positive	1 (0.8%)	12 (10.6%)	7 (6.2%)
True-negative	92 (81.4%)	46 (40.7%)	18 (15.9%)
False-negative	10 (8.9%)	13 (11.5%)	4 (3.5%)
Area under the curve (95% CI)	0.87 (0.78–0.95)	0.87 (0.81–0.93)	0.95 (0.90–1.00)
Sensitivity (95% CI)^a^	50.0% (27.2–72.8)	76.4% (63.0–86.8)	95.5% (88.8–98.7)
Specificity (95% CI)^a^	98.9% (94.2–100.0)	79.3% (66.6–88.8)	72.0% (50.6–87.9)
PPV (95% CI)^a^	90.9% (58.7–99.8)	77.8% (64.4–88.0)	92.3% (84.8–96.9)
NPV (95% CI)^a^	90.2% (82.7–95.2)	78.0% (65.3–87.7)	81.8% (59.7–94.8)

CI, confidence interval; PPV, positive predictive value; NPV, negative predictive value.

a Binary classifier using a 0.5 predicted probability threshold for each model.

### Sensitivity analyses

Four sensitivity analyses were conducted in each of the three primary models predicting 10-, 30- and 100-day mortality. Two variables not initially selected for inclusion had borderline BIF (25–49%). These were EEG and CSF s100b abnormality, with BIF of 43.5 and 40.6%, respectively. We therefore estimated the primary models including these variables separately, and together, and used AUC to compare overall model performance with the primary model. As a fourth sensitivity analysis, we compared the overall model performance with and without *PRNP* codon 129 genotype. This variable was initially included in the final models, although results confirming *PRNP* codon 129 genotype are not currently available immediately, and so assessing the contribution of this variable to model performance could inform the importance of expediting these results. As shown in Table 4, none of the primary models was improved by the addition of EEG abnormality, CSF s100b abnormality or both variables together. Exclusion of *PRNP* codon 129 genotype significantly reduced performance of models predicting 30- and 100-day mortality (although the models still performed well, AUC = 0.90 and 0.89, respectively).

**Table 4:** Sensitivity analyses comparing the area under the curve of primary models and alternative models in predicting 10-, 30- and 100-day mortality in sCJD patients

	10-day mortality AUC (95% CI)	30-day mortality AUC (95% CI)	100-day mortality AUC (95% CI)
Primary model	0.94 (0.92–0.96)	0.92 (0.90–0.94)	0.91 (0.89–0.94)
Primary model plus EEG abnormality	0.94 (0.92–0.96), *P* = 0.152	0.92 (0.90–0.94), *P* = 0.282	0.91 (0.89–0.94), *P* = 0.061
Primary model plus CSF s100b abnormality	0.94 (0.92–0.96), *P* = 0.450	0.92 (0.90–0.94), *P* = 0.313	0.91 (0.89–0.94), *P* = 0.675
Primary model plus EEG and CSF s100b abnormality	0.94 (0.92–0.97), *P* = 0.073	0.92 (0.90–0.94), *P* = 0.498	0.91 (0.89–0.94), *P* = 0.439
Primary model without *PRNP* codon 129 genotype	0.94 (0.91–0.96), *P* = 0.190	0.90 (0.88–0.93), *P* < 0.001	0.89 (0.86–0.92), *P* = 0.001

AUC, area under the curve.

## Discussion

We have developed accurate prognostic models for survival and care status escalation in sCJD patients using data routinely available in specialist diagnostic services. We observed highly heterogenous outcomes for both survival and care status, reinforcing the need for accurate prognostic decision-making aids to enhance clinical trials and care management. We investigated three main types of predictors. First, measurement of the degree of disease progression at the time of initial assessment. There is no perfect rating scale for disease progression; we considered our previously developed scales for sCJD (the MRC Prion Disease Rating Scale, and Cognitive and Motor Examination Scales); the MMSE, EEG abnormality, clinical category of sCJD and care status. Second, prediction of the rate of change of disease progression, for which we investigated the MRI brain scan, *PRNP* codon 129 genotype, CSF biomarkers and time since disease onset. Third, we included patient characteristics (age and sex).

Our primary models predicting survival can be compared with that developed by Llorens *et al*.,^18^ which used data from the German Reference Centre for Transmissible Spongiform Encephalopathies to predict 6-month survival from sCJD onset. Both studies used data from national specialist diagnostic services with similar patient age and sex. The Llorens model of total survival from symptom onset incorporated age, sex, *PRNP* codon 129 genotype and CSF tau with moderate accuracy (AUC = 0.69; 95% CI = 0.67–0.71); our models achieved greater performance (AUC 0.91–0.94). We predicted outcomes over a shorter period (10, 30 and 100 days) from date of diagnosis, a more precise time measurement than carer-reported symptom onset, which is typically a retrospective estimation, and more useful from the perspective of clinical decision making. Both models included sex and *PRNP* codon 129 genotype. Llorens *et al.* also included age and CSF tau levels, whereas we included a wider range of additional predictors (days since symptom onset, baseline care status, MRC Prion Disease Rating, Motor and Cognitive Scores, MRI abnormalities, MMSE score and clinical phenotype). The improved performance may therefore be in part due to predicting from diagnosis rather than symptom onset, and the wider range of predictors incorporated in the current study.

Our study has some limitations. Given the rarity of sCJD the sample size is large, though from a modelling perspective it is moderate. An external dataset for model validation was not available, and we therefore used leave-one-out cross-validation. The sample was particularly limited for patients with early disease and minimal care status, reflecting the difficulty in prompt diagnosis of a rapidly progressive rare disease. Nevertheless, to our knowledge this is the first attempt to prognosticate care status. Due to the limited sample for this secondary subgroup analysis, we did not repeat the variable selection procedure, and instead developed models using the predictors selected for the primary survival models. The pool of potential predictors was determined by what is typically available in the UK at the point of initial diagnosis. Other predictors of potential value, for example serum NfL, serum total tau, CSF total tau and other biomarkers and more sophisticated imaging metrics, were generally only available in the UK as research tests during the period of this study, and were therefore not investigated. As CSF total tau and serum NfL are becoming increasingly available in routine clinical practice in the UK, these may be helpful additions in future prognostic modelling. A wealth of evidence points to the role of prion strain, or sCJD subtype, in determining clinical phenotype. Unfortunately, there are no direct biomarkers of CJD subtype that can be used in life, although the recent development of an imaging method to infer subtype may prove useful in the future.^10^ Further exploration of how to incorporate different imaging abnormalities (e.g. atrophy, regions of diffusion abnormality), rather than a simple count, are warranted. As these biofluid and imaging biomarkers move into routine clinical use, they could be evaluated for improving model performance.

Because the apparent annual incidence of sCJD has steadily increased over the last 30 years, it is likely that the disease has not been fully ascertained. The Cohort study did not recruit all patients documented as dying from sCJD in the study period, because either the diagnosis was not made in life, the patient died before we were able to visit, or after 2015, was too advanced in disease stage to be eligible, or the patient or family did not agree to join. It is therefore likely that the study is not fully representative of the theoretical totality of the disease, with diminished ascertainment of the very fastest progressing patients, and those with atypical phenotypes that make the disease hard to diagnose. The secondary outcome of care status was defined in a pragmatic way based on our observations about how care is typically provided to patients with sCJD in the UK. Experiences in other countries may differ, for example regarding the timing and use of formal 24-h care facilities. Furthermore, some variability in care status may potentially be influenced by factors other than symptom severity, for example socioeconomic factors, and patient or caregiver preferences. Future studies might examine the degree to which models like the one presented here generalize to other countries and populations, with careful consideration about how care status is defined in consideration of local practices. Also, with respect to outcomes, ‘time to akinetic mutism (e.g. MRC Scale <3)’ or ‘time to death, mechanical ventilation or tube feeding’ could be valuable to explore in future studies, and might be predicted more accurately than survival per se.

We should be cautious in assuming that prognostic information about likely survival and care status will necessarily be beneficial to patients and carers, who may have differing views on whether this information is welcome.^28^ Indeed, it may even cause harm if this information is shared without careful explanation and compassion. Further pragmatic studies, which facilitate clinical implementation of such models are essential. We envisage prospective evaluations would still require specialist interpretation of test results, for example of MRI scan findings, which we know are not reliably reported locally. Key outcomes include usability and acceptability to clinicians, patients and carers, and whether the provision of information leads to improved patient outcomes and care provision.

There have been few clinical trials in sCJD relative to other dementias. In part, this relates to the rarity of the disease and the challenges evident from rapid progression. Two experimental treatments have currently either entered human use, or are planned for human use: monoclonal antibody therapy^29^ and antisense oligonucleotide treatments. The mechanism of actions may require time for the compound to achieve target therapeutic levels in brain tissues, and clinical trials may be less powerful if patients are recruited with a very short prognosis. Prognostic modelling may therefore have value in deciding on eligibility for future clinical trials.

## Conclusions

Among sCJD patients attending a specialist diagnostic service, routinely collected clinical, imaging and genetic data can be used in combination to accurately predict survival and escalating care status. This has the potential to enhance prognostic decision making during clinical practice and to facilitate the timely provision of support to patients and their families.

## Supplementary Material

fcac201_Supplementary_Data

## Abbreviations

AUC =: areas under the curve
BE =: backwards elimination
BIF =: bootstrap inclusion fraction
FSP =: function selection procedure
IQR =: interquartile range
MMSE =: Mini-Mental State Examination
MRC =: Medical Research Council
NfL =: neurofilament light
NPV =: negative predictive value
PPV =: positive predictive value
PSWCs =: Periodic Sharp Wave Complexes
ROC =: receiver operating characteristic
sCJD =: sporadic Creutzfeldt-Jakob disease
